# Maternal and perinatal health in undocumented migrants: estimating access and outcomes through HMIS

**DOI:** 10.1093/eurpub/ckac131.527

**Published:** 2022-10-25

**Authors:** E Genovese, A Cantarutti, G Corrao, A Locatelli

**Affiliations:** 1 Department of Medicine and Surgery, University of Milan-Bicocca, Milan, Italy; 2 Department of Statistics, University of Milan-Bicocca, Milan, Italy

## Abstract

**Background:**

Vulnerability and inequality are exacerbated in undocumented migrants, the most invisible to health systems.

**Objectives:**

To estimate maternal and perinatal health needs in undocumented migrants and test a methodology for systematic monitoring & evaluation.

**Methods:**

Population-based retrospective cohort study based on routine data through maternity records and temporary registration code in a sub-national Health Management Information System.

**Results:**

420924 deliveries including 1524 undocumented migrants having accessed maternity care through the NHS in Lombardy Region (Italy) from 2016 to 2021 were included. Demographics and social determinants: undocumented migrants were born in Europe (non-EU) (36%), Americas (30%), Africa (6%), Western Pacific (3%), South-East Asia (2%), Italy (2%), were stateless (7%); 52% had no/low schooling, 92% were unemployed and 52% non-married, compared to 15%, 20%, and 44% Italians. Obstetric history and antenatal care: 22% undocumented migrants had a previous abortion and 15% a previous cesarean delivery; 58% had ≥5 antenatal visits, 67% first ANC visit in trimester 1, 64% ≥ 2 ultrasounds incl. first in trimester 1, 6% full laboratory tests, compared to 90%, 97%, 97%, and 66% Italians. Intra-partum and perinatal care: 45% undocumented migrants delivered in a public hospital with neonatal intensive care unit; 69% had a normal delivery, 5% instrumental delivery, 10% and 27% emergency and total cesarean section; 2.6% neonates had emergency resuscitation and 49% were breastfed <2h from birth. Outcomes: 81% physiological pregnancies, 2.3% severe hemorrhage, 4.8% intra-uterine growth retardation, 9.3% pre-term delivery, 17% small for gestational age, 7% low birth weight, 0.6% poor Apgar score, 3% malformations.

**Conclusions:**

Maternal and perinatal health was poor in undocumented migrants, varying by birthplace. Social determinants, health coverage and outcomes showed vulnerability and inequality compared to the general population.

**Key messages:**

• Tailored interventions are needed: outreach health promotion on safe motherhood and neonatal care, healthcare provider training, cultural mediation, translation, and functional language learning.

• A systematic monitoring and evaluation system needs to routinely collect, integrate, and analyze data on key indicators.

